# Nerve Fibers in the Tumor Microenvironment Are Co-Localized with Lymphoid Aggregates in Pancreatic Cancer

**DOI:** 10.3390/jcm10030490

**Published:** 2021-01-30

**Authors:** Lara R. Heij, Xiuxiang Tan, Jakob N. Kather, Jan M. Niehues, Shivan Sivakumar, Nicole Heussen, Gregory van der Kroft, Steven W. M. Olde Damink, Sven Lang, Merel R. Aberle, Tom Luedde, Nadine T. Gaisa, Jan Bednarsch, Drolaiz H. W. Liu, Jack P. M. Cleutjens, Dominik P. Modest, Ulf P. Neumann, Georg J. Wiltberger

**Affiliations:** 1 Department of General, Gastrointestinal, Hepatobiliary and Transplant Surgery, RWTH Aachen University Hospital, 52074 Aachen, Germany; xtan@ukaachen.de (X.T.); gvanderkroft@ukaachen.de (G.v.d.K.); steven.oldedamink@maastrichtuniversity.nl (S.W.M.O.D.); svlang@ukaachen.de (S.L.); m.aberle@maastrichtuniversity.nl (M.R.A.); jbednarsch@ukaachen.de (J.B.); uneumann@ukaachen.de (U.P.N.); gwiltberger@ukaachen.de (G.J.W.); 2 NUTRIM School of Nutrition and Translational Research in Metabolism, Maastricht University, 6229 ER Maastricht, The Netherlands; 3 Institute of Pathology, RWTH Aachen University, 52074 Aachen, Germany; ngaisa@ukaachen.de; 4 Department of Medicine III, University Hospital RWTH Aachen, 52074 Aachen, Germany; jkather@ukaachen.de (J.N.K.); jan.niehues@rwth-aachen.de (J.M.N.); tluedde@ukaachen.de (T.L.); 5 Department of Oncology, University of Oxford, Oxford OX3 7DQ, UK; shivan.sivakumar@oncology.ox.ac.uk; 6 Kennedy Institute of Rheumatology, University of Oxford, Oxford OX3 7FY, UK; 7 Department of Medical Statistics, RWTH Aachen University, 52074 Aachen, Germany; nheussen@ukaachen.de; 8 Center of Biostatistics and Epidemiology, Medical School, Sigmund Freud University, 1020 Vienna, Austria; 9 Department of Surgery, Maastricht University Medical Center, 6229 HX Maastricht, The Netherlands; 10 Department of Pathology, Maastricht University Medical Center, 6229 HX Maastricht, The Netherlands; drolaiz.liu@mumc.nl (D.H.W.L.); jack.cleutjens@mumc.nl (J.P.M.C.); 11 CARIM Cardiovascular Research Institute Maastricht, Maastricht University, 6229 HX Maastricht, The Netherlands; 12 Department of Hematology, Oncology and Tumor Immunology, CVK, Charité Universitätsmedizin Berlin, 13353 Berlin, Germany; dominik.modest@charite.de

**Keywords:** tumor microenvironment, machine learning, nerve fiber density, spatial arrangements, tertiary lymphoid structures, pancreatic cancer

## Abstract

B cells and tertiary lymphoid structures (TLS) are reported to be important in survival in cancer. Pancreatic Cancer (PDAC) is one of the most lethal cancer types, and currently, it is the seventh leading cause of cancer-related death worldwide. A better understanding of tumor biology is pivotal to improve clinical outcome. The desmoplastic stroma is a complex system in which crosstalk takes place between cancer-associated fibroblasts, immune cells and cancer cells. Indirect and direct cellular interactions within the tumor microenvironment (TME) drive key processes such as tumor progression, metastasis formation and treatment resistance. In order to understand the aggressiveness of PDAC and its resistance to therapeutics, the TME needs to be further unraveled. There are some limited data about the influence of nerve fibers on cancer progression. Here we show that small nerve fibers are located at lymphoid aggregates in PDAC. This unravels future pathways and has potential to improve clinical outcome by a rational development of new therapeutic strategies.

## 1. Introduction

The incidence of Pancreatic Ductal Adenocarcinoma (PDAC) almost equals the mortality rate illustrating the aggressiveness of PDAC [1]. In order to understand the resistance to therapeutics the components within the cancer stroma need to be further unraveled. PDAC arises in a tumor microenvironment (TME) that is characterized by extensive communication between tumor cells and non-malignant cells. Specifically, stromal components have been mechanistically implied in immune evasion in the context of cancer therapy, including immunotherapy [2,3]. The role of nerve fibers within the cancer-associated stroma has not been fully investigated. The aim of this study is to define the role of small nerve fibers in the cancer stroma and their spatial arrangement to immune cells. We hypothesized that small nerve fibers are one of the key components in cancer progression.

Recent publications show that B cells play an important role in the survival of cancer patients and response to immunotherapy [4,5,6,7,8], and this is not limited to T cells only [9,10,11]. Furthermore, B cells play an important role in the tumor formation of PDAC [12]. Tertiary lymphoid structures (TLS) have been recognized as ectopic lymphoid organs that reside in inflamed tissue and also in cancer [13,14]. These structures show differences in the maturation stage and sometimes result in the formation of a germinal center [14,15,16]. The different maturation stages of TLS show expression of different cells: early TLS without the formation of a germinal center, primary follicle-TLS with CD21 positive follicular dendritic cells without the formation of a germinal center and secondary follicle-TLS with the presence of a germinal center (also CD23-positive) [17]. TLS presence is described in multiple cancer types and is variably present in cancer types and patients and is a favorable prognostic factor [18,19].

## 2. Methods

### 2.1. Ethics Statement

All experiments were conducted in accordance with the Declaration of Helsinki and the International Ethical Guidelines for Biomedical Research Involving Human Subjects. Pathology blocks from the University Hospital of Aachen (RWTH Aachen) were retrieved (institutional ethics EK 106/18). Written human patient consent was not necessary, because this study was based on retrospective chart review and archived pathology material. Nevertheless, most patients had signed informed consent; in some cases, informed consent was waived due to the lack of risk for the patient and the fact that those patients were unable to provide informed consent. Images from tissue specimens are entirely unidentifiable, and no details on individuals are reported within the manuscript.

#### 2.1.1. Patient Cohort

Histological slides from 166 patients with PDAC were selected and used to cut tissue sections for the immunostaining Protein Gene Product 9.5 (PGP9.5).

Of the 188 patients included in the cohort, 15 were excluded due to in-hospital mortality, 3 due to a loss to follow up and 4 due to poor-quality pathology slides. Analysis was performed on 166 cases. Clinical data for this cohort are listed in Table 1.

#### 2.1.2. Pathological Examination

The clinic-pathological parameters, including tumor size, differentiation, positive lymph node status and R0/R1, were carefully reviewed in the original report. All PDAC lesions were pathologically examined and classified according to World Health Organization (WHO) classification using the 8th edition of the Tumor Nodes Metastasis (TNM) Classification for Malignant Tumors.

### 2.2. Nerve Fiber Density (NFD)

Immunohistochemistry was performed on formalin-fixed, paraffin-embedded tissue sections. Sections (2.5 μm thick) were cut, deparaffinized in xylene and rehydrated in graded alcohols. Slides were boiled in citrate buffer (pH 6.0) at 95–100 °C for 5 min and were cooled for 20 min and endogenous peroxide in methanol for 10 min. Sections were incubated with rabbit anti-human PGP 9.5 (DAKO 1:100) overnight at 4 °C.

A single digital image was uploaded in Qupath 0.1.6, which is a flexible software platform suitable for a range of digital pathology applications. Nerve fiber density (NFD) was evaluated by counting the number of nerve fascicles with diameters of <100 μm in 20 continuous fields at × 200 magnification.

Nerve fiber density results were grouped into 3 categories: (1) negative, no nerve fibers, (2) weak expression, 1–10 nerve fibers and (3) moderate/strong expression >10 nerve fibers, according to the existing literature on breast cancer [20].

### 2.3. Tumor Cellularity (TC)

Every immunostained slide was scanned, and with whole slide imaging, the tumor glands, normal pancreatic tissue and atrophic pancreatic tissue were manually annotated in QuPath 0.1.6 by a senior pathologist [18]. The stromal area was measured by the following formula: total tissue-(normal tissue + atrophic pancreas + tumor) = stroma surface. Tumor cellularity was measured as (tumor surface/(tumor surface + stroma surface).

### 2.4. Phenotyping of Immune Cells

Based on H&E, the predominant type of immune cells was judged manually by the PhD student and the senior pathologist. The H&E slide and the slide used for immunostaining were cut directly after each other. The dominant type of immune cells was determined and manually scored into three categories: (1) lymphocyte-predominant, (2) neutrophil-predominant or (3) no immune cells.

### 2.5. Single Immunohistochemistry

To further define the type of immune cell, we performed immunohistochemistry on 10 patients with histologically confirmed lymphoid aggregates on H&E staining and a high NFD based on the PGP9.5 staining. We used CD20 (B cells), CD4, CD8 (T cell markers) and FOXP3 (which can be expressed by T cells), according to previous literature that CD20+ B cells were located in the TLS and were colocalized with CD4+, CD8+ and FOXP3+ T cells [6]. To illustrate the presence of follicular dendritic cells, we also stained for CD21. These stainings were compared to the H&E routine staining and PGP9.5 nerve fiber staining.

Sections were stained with mouse or rabbit anti-human monoclonal antibodies against CD20 (Dako, L26, 1:200), CD21 (DAKO, 1:25), CD23 (Leica, CD23-1B12, 1:50), CD4 (DAKO, 4B12 1:50), CD8 (DAKO, C8/144B, 1:50) and FOXP3 (DAKO, PCH101, 1:50). All sections were counterstained with hematoxylin, dehydrated and mounted. All sections were cover-slipped using Vectashield Hardset 1500 mounting medium with DAPI, and slides were scanned and digitalized using the Roche Ventana scanner. Immunohistochemistry staining was interpreted in conjunction with H&E-stained sections.

### 2.6. Multiplex Immunofluorescence Assay and Analysis

For immunofluorescence multiplex staining, we followed the staining method for the following markers: CD20 (Dako, L26, 1:500) with subsequent visualization using fluorescein Cy3 (1:100); FOXP3 (DAKO, PCH101, 1:300) with subsequent visualization using fluorescein FITC (1:100); PGP9.5 (DAKO 1:300) with subsequent visualization using fluorescein TEX RED (1:100) and nuclei visualized with DAPI.

**Figure 1:**
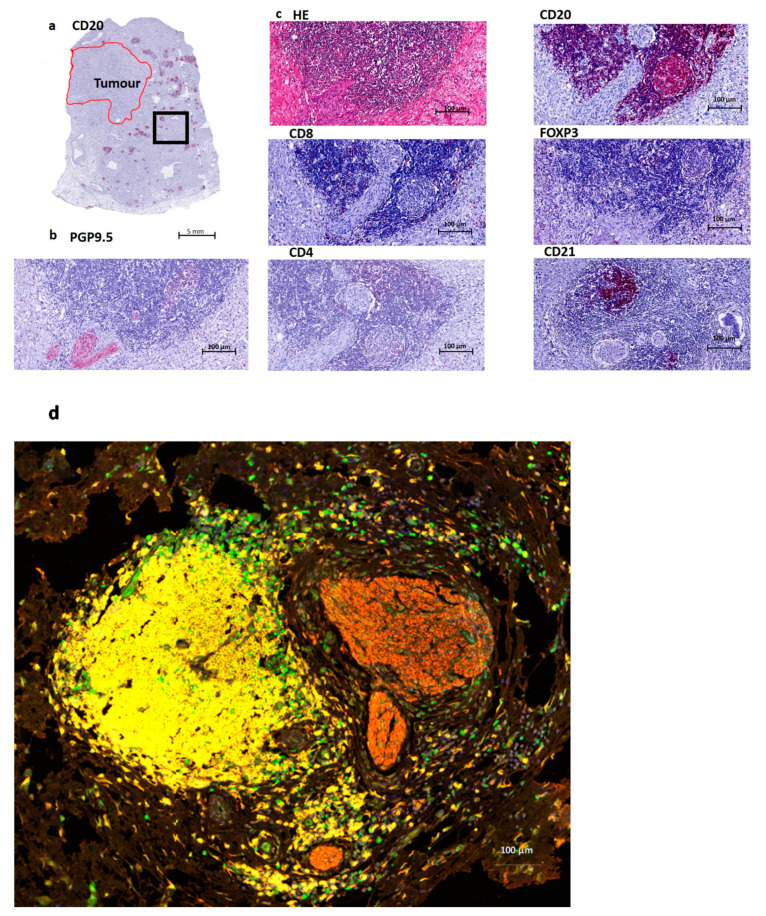
Overview of a tertiary lymphoid structure (TLS) in Pancreatic Cancer. (**a**). Overview of a CD20 staining (B cells) in a Pancreatic Ductal Adenocarcinoma (PDAC) patient. The red annotated area indicates the tumor region, and the black box indicates the area of magnification for Figure 1b–d. (**b**). Nerve fiber staining PGP9.5, which is a pan-neuronal marker. This image shows the presence of nerve fibers at the edge of a lymphoid aggregate. (**c**). Routine HE staining containing B cells (CD20), T cells (here mostly CD8 and CD4) and follicular dendritic cells (CD21). Treg cells (FOXP3) are mostly absent, just as CD4 in this image. CD8 shows a few positive cells at the border of this structure. (**d**) Multiplex imaging: T cells (FOXP3 green), B cells (CD20 yellow), Nerves (PGP9.5 red) and nucleus (DAPI blue).

The slides were scanned using the TissueFAXS slide scanner (supplier TissueGnostics). For each marker, the mean fluorescent intensity per case was then determined as a base point from which positive cells could be established. For multispectral analysis, each of the individually stained sections was used to establish the spectral library of the fluorophores. The senior pathologist selected the Region of Interest (ROI) at 20× magnification. See Figure 1 for an overview of the immunostainins performed. 

### 2.7. Lymphoid Aggregate Count Using Machine Learning

**Figure 2:**
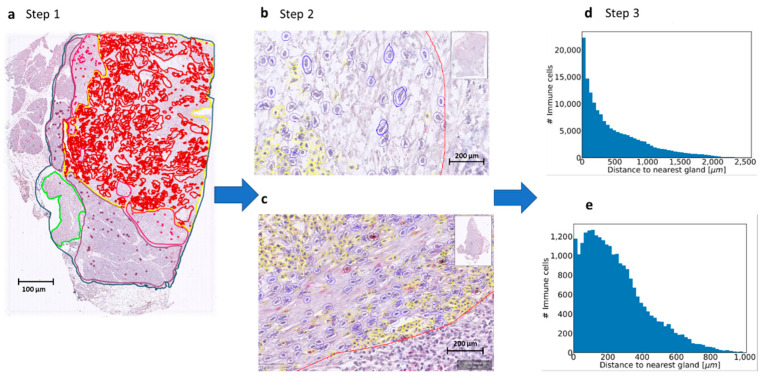
Process using machine learning to determine immune cell spatiality. (**a**). Step 1. Defining the Region of Interest (ROI) (yellow line). Every scanned slide was annotated in: total tissue (dark blue), normal pancreas (purple), atrophic pancreas (pink), normal duodenum (green) and tumor (red). For the machine learning classifier, a Region of Interest (ROI) was also annotated (yellow). In this area, only the cell detection classifier was used to detect the stromal cells in fibroblasts and immune cells. (**b**,**c**). Step 2. The cell classifier was trained by the pathologist to recognize fibroblasts (blue annotations) and immune cells (yellow annotations). The tumor glands were all annotated manually gland by gland and are shown in red annotation. (**d**,**e**). Step 3. Measurement of distance of immune cell to tumor gland. From each slide, a plot was made with the number of immune cells (*y*-axis) and the distance to the nearest tumor gland (*x*-axis). These plots were used to measure the mean distance from the immune cell to the tumor gland in micrometers. Some slides showed immune cells at a greater distance from the tumor and some slides showed immune cells nearby the tumor. With this technique, we could identify immune cell aggregates, so groups of immune cells were located close to each other.

By using the annotations on the immunostained slide (PGP9.5), the distance from each immune cell to the tumor gland was measured, see Figure 2. The spatial arrangement of the immune cells was determined by a semi-automated machine learning workflow, which comprised cell segmentation, feature computation and stroma- and immune cell identification. To facilitate high throughput of thousands of immune cells on multiple images, QuPath enables interactive training of cell classification, after which the classifier can be saved and run over multiple slides. The senior pathologist trained a cell detection classifier to recognize immune cells and fibroblasts in a certain Region of Interest (ROI). This ROI was annotated by the senior pathologist and contained tumor glands and stroma only; including normal tissue and/or atrophic tissue was avoided in order to achieve the best detection results. Application of this workflow resulted in both fine-grained cell-by-cell analysis and overall summary scores of the spatial arrangements of the immune cells within the ROI in relation to the annotated tumor glands. The results are visualized via color-coded markup images.

**Figure 3:**
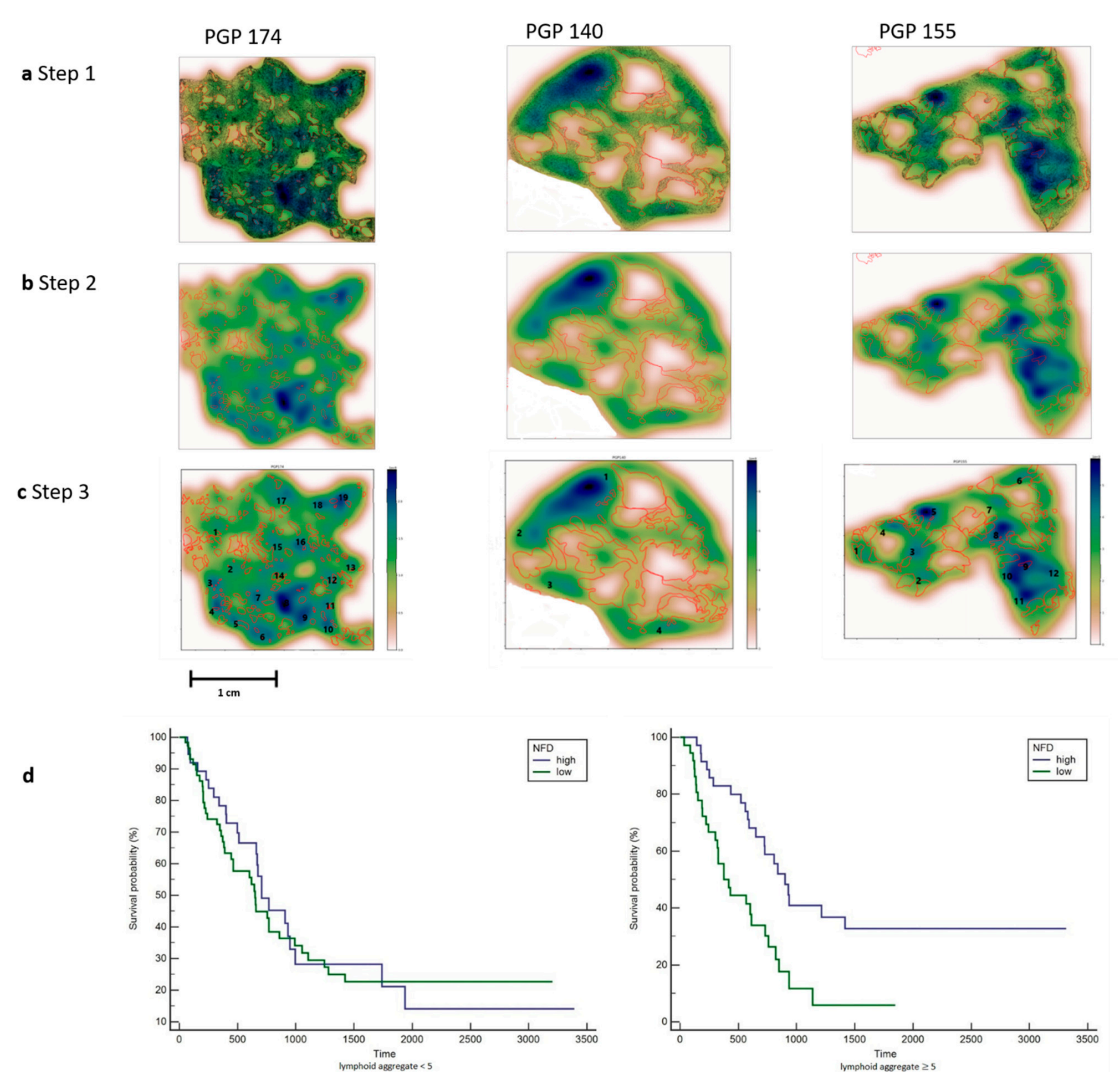
Counting the number of lymphoid aggregates in patients with Pancreatic Cancer. (**a**). Step 1. All immune cells are plotted using the x and y coordinates. Tumor glands are annotated in red. Three patients are shown with a different distribution pattern of the immune cells. PGP 174 is immune-cell-rich and shows aggregates at the edge and in between the tumor glands. PGP 140 only shows a few immune cell aggregates at the edge of the tumor. PGP 155 shows a few immune cell aggregates mainly located at the edge of the tumor. (**b**). Step 2. A heat map was created by using a 2D Kernel Density. The color blue was used because of the already red annotations for tumor glands. Dark blue areas show a high density of immune cells. (**c**). Step 3. The heat map clusters with a dark blue and light blue color were interpreted as lymphoid aggregates and counted manually. Each number corresponds with a lymphoid aggregate, images with many lymphoid aggregates (PGP 174) and only few lymphoid aggregates (PGP140) are shown. (**d**). Left: Kaplan–Meier plot for patients with less than 5 lymphoid aggregates show no significance in survival. Right: Kaplan–Meier plot for patients with 5 or more Lymphoid Aggregates and a high NFD show a significantly better survival.

To obtain estimates for immune cell densities, a kernel-density estimate using Gaussian kernels as implemented in the gaussian_kde class from SciPy was applied. First, kernels were fitted from immune cell positions and calculated for points on a 100 × 100 grid for each slide. The results are displayed as a heat map together with the slides tumor annotations (see Figure 3). From this heat map, regions of high immune cell density can be identified manually that correspond to a lymphoid aggregate.

### 2.8. Statistics

Continuous variables were summarized by means and corresponding standard deviations (SD). Categorical data were presented by frequencies and percentages. Cox regression models were used to analyze the joint relation between clinical variables (coded by 0 and 1 for binary variables) on overall mortality. All exploratory variables were studied in a univariate Cox regression model adjusted for age, gender and BMI. Exploratory variables were assessed as relevant to be mutually included in our final model if the *p*-value was below 0.05. Relevant variables were studied further for pairwise interaction. In doing so, we used again the significance margin of 5%. Then a multivariate Cox regression model adjusted for age, gender and BMI with backward selection was fitted to the previously identified variables and interactions. During this final step, the significance level for removing a variable or interaction from the model was set to 0.05. For the final Cox model, graphical and numerical methods according to Lin et al. were performed to establish the validity of the proportionality assumption [21]. No deviation from the model assumption could be observed. We report our results by hazard ratios, corresponding 95% confidence limits and *p*-values, where a *p*-value of less than or equal to 0.05 could be interpreted as statistically significant test results. Forest plots were chosen for graphical visualization, and Kaplan–Meier plots for comparison of subgroups. All analyses were performed using SAS^®^ statistical software, V9.4 (SAS Institute, Cary, NC, USA).

## 3. Results

### 3.1. High Nerve Fiber Density Is Associated with a Better Survival in Pancreatic Cancer

To gain deeper insight into the influence of nerve fibers on survival we used the neuronal immunohistochemistry staining PGP9.5 on our cohort of patients with pancreatic cancer (n = 166). A multivariate Cox regression model adjusting for age, gender and BMI revealed high Nerve Fiber Density (NFD) (more than 10 positive nerve fibers with diameters of <100 μm in 20 continuous fields at × 200 magnification) to be associated significantly with prolonged overall survival (HR 1.676 (95%CI 1.126, 2.495) for low vs. high NFD, *p*-value 0.0109) as compared to low NFD. We have used small nerve fiber innervation of the blood vessels in the normal tissue as an internal positive control in case of a low-NFD tumor (see Supplementary Figures S1 and S2).

### 3.2. The Lymphozcyte Predominant Immunophenotype Mainly in a Low Cellular Tumor

To further understand to what extent immune cells and nerve fibers may interact, each routine H&E slide was scored manually into a lymphocyte-predominant, neutrophil-predominant or immune-cell-depleted phenotype [22]. The scoring was done by a senior pathologist and the Ph.D. and based on the most dominant immune cell on one tumor slide only by viewing the histology on HE staining. In this evaluation, the abundant stromal phenotype, i.e., a low cellular tumor, was associated with the lymphocyte-predominant phenotype. High tumor cellularity was significantly associated with poor survival (HR 4.287 (95%CI 1.460, 12.589), *p*-value 0.0081 for one unit increase). All results are summarized in Table 1.

### 3.3. Immune Cells Located at the Nerve Fibers Are Mainly B Cells

Single immunohistochemistry was used to define which immune cells are co-localized with the nerve fibers. The immune cells that presented in the direct surroundings of the nerve fibers were mainly CD20-positive. The definition of TLS differs based on the maturation stage, but it is commonly accepted that TLS are composed of a B cell zone and a T cell zone and may show the presence of a germinal center. To evaluate the maturity of the TLS, we used CD21 to define follicular dendritic cells. In our cohort, we found clusters of B lymphocytes with and without a T cell zone and follicular dendritic cells corresponding to different maturation stages of TLS. Multiplex immunofluorescence shows the architecture of a TLS in Pancreatic Cancer (see Figure 1), and this image provides an overview of the distribution of the immune cell aggregates at the edge of the tumor.

### 3.4. Machine Learning for Quantification of Lymphoid Aggregates

To gain insight into the spatial arrangements of the immune cells, we used machine learning to further measure the distance from the immune cells to the tumor glands. The slides were first scanned, and annotations were made in QuPath 0.1.6. A cell detection classifier was trained to recognize immune cells within a region of interest and to separate the immune cells from fibroblasts (see Figure 2). To obtain estimates for immune cell densities, a kernel-density was applied. The results are displayed as a heat map together with the slides tumor annotations (see Figure 3). To examine the predictive power of the number of lymphoid aggregates (LA) in combination with nerve fiber density and tumor cellularity on overall survival, an additional exploratory Cox regression model with LA, NFD and TC, as well as the interaction between LA and NFD or TC respectively was evaluated. As the interaction between LA and TC was non-significant at a 5% level, the interaction was removed from the model. Thus, the exploratory model contains LA, NFD, TC and the interaction between LA and NFD as explanatory factors. The significant NFD*LA interaction (*p*-value 0.0220) suggests that the effect of NFD is different by LA. For LA, number greater or equal to 5 mortality is significantly lower in patients with high NFD compared to patients with low NFD (20% (n = 14) vs. 10% (n = 7); HR 0.388 (95%CI 0.218, 0.689)), whereas for LA number less than 5, no significant difference between patients with high or low NFD could be shown (14% (n = 13) vs. 19% (n = 18); HR 0.959 (95%CI 0.573, 1.604)), which is also apparent in the corresponding Kaplan–Meier plot (See Figure 3).

## 4. Discussion

PDAC is known for its significant cancer-associated stroma or so-called tumor microenvironment. The large stromal component is a significant area of investigation and is held responsible for poor treatment response. In this study, we show that nerve fibers also play a role in the TME. Nerves are emerging regulators of cancer initiation, progression, and metastasis [23]. Previous data described by Renz et al. suggest that cholinergic signaling by the parasympathetic nerves can suppress the growth of pancreatic cancer cells, where the sympathetic nerves stimulate the growth of pancreatic cells. Therefore, in pancreatic cancer cells, there is a balance of neural influence [24]. Immune cells also play a role in nerves in cancer and are a potential target. There are many levels of neuroimmune interactions, including regulation of inflammation, that play a role in cancer growth and dissemination [25].

Neural invasion by tumor cells is one of the most striking characteristics of PDAC and is a sign of aggressive behavior. Surprisingly, in this study, we found that a high NFD is associated with better survival in patients with PDAC. We observed perineural tumor invasion of the bigger nerve trunks, and staining was positive in PGP9.5, but these nerves were not counted due to exclusion based on their size. The smaller nerve fibers were not associated with tumor invasion, and these nerve fibers were included in the counting method for NFD. Therefore, NFD is determined as the number of small nerve fibers, not to be confused by nerves, invaded by tumor cells. We found that these small nerve fibers are located around lymphoid aggregates and predict a better survival in patients with five or more lymphoid aggregates and a high NFD. The definition of TLS differs in the literature depending on the maturation stage. The presence or absence of a germinal center shows different maturation stages of the TLS [15]. Mature TLS show the presence of germinal centers with the expression of CD21 and CD23. With the use of machine learning, we could only be certain of the presence of lymphoid aggregates and to specify these aggregates as real TLS, further immunohistochemistry is needed.

PDAC is known for its tumor microenvironment, and here, cross-talk takes place between tumor and host. The immune environment is influenced by the tumor and the host. The immune system is known to have a crucial role in cancer, and this is possibly regulated by genetic and morphological features of the tumor. It is known that PDAC patients with higher levels of CD4+ and CD8+ T cells have a better survival [26]. Surrounding stromal cells support tumor budding of the cancer cells and promote aggressive behavior; it is described that this phenotype contains a depletion of TILs [27]. The presence of tumor-infiltrating lymphocytes (TILs) is a predictor of a better prognosis. In breast and ovarian cancer, a major component of TILs is the tumor-infiltrating lymphocytic B cells (TIL-B) [28]. In our study, we show many B-cells at the edge of the tumor. Previous literature has shown that B cells and tertiary lymphoid structures promote immunotherapy response in melanoma and sarcoma [4,5,6] and are of main importance for better survival. The role of nerve fibers in the co-localization with these lymphoid aggregates has the potential to discover new pathways for a better survival and it paves the way for new possible targets for (combination) therapy. We expect that these data may be broadly applicable to other malignancies.

## Figures and Tables

**Table 1:** Results from univariate and multivariate Cox regression analysis adjusted for age, gender and BMI of prognostic factors associated with overall survival.

		Univariate Analyses (Adjusted for Age, Gender, BMI)		Multivariate Analysis (Adjusted for Age, Gender, BMI)	
Variable	Mean (SD) or Frequency (%)	Hazard Ratio [95%-C]	*p*-Value	Hazard Ratio [95%-CI]	*p*-Value
Age	66 (10)			1.024 [1.002, 1.047]	0.0356
Gender female	80 (48.19)			1.173 [0.789, 1.745]	0.4297
male	86 (51.81)				
BMI	25.7 (4.3)			0.996 [0.954, 1.040]	0.8547
ASA <3	62 (37.35)	0.909 [0.607, 1.362]	0.6449		
≥3	104 (62.65)				
Tumour grade G2	94 (56.63)	1.954 [1.342, 2.844]	0.0005		
G3	72 (56.63)				
Extent of tumour T1/T2	26 (15.66)	2.116 [1.015, 4.410]	0.0455		
T3/T4	140 (84.34)				
Perineural invasion Absent	28 (16.87)	2.239 [1.265, 3.961]	0.0056	2.409 [1.337, 4.340]	0.0034
Present	138 (83.13)				
Lymph node metastasis Absent	39 (23.49)	2.322 [1.407, 3.834]	0.0010		
Present	127 (76.51)				
Lymphatic invasion Absent	114 (68.67)	2.080 [0.418, 3.050]	0.0002	1.763 [1.173, 2.651]	0.0064
Present	52 (31.33)				
Venous invasion Absent	136 (81.93)	1.452 [0.923, 2.284]	0.1068		
Present	30 (81.93)				
Surgical margin status Negative	106 (63.86)	1.962 [1.342, 2.868]	0.0005		
Positive	60 (36.14)				
Nerve fiber density High	72 (43.37)	1.597 [1.093, 2.336]	0.0155	1.676 [1.126, 2.495]	0.0109
Low	94 (56.63)				
Lymphoid Aggregates <5	95 (57.23)	1.084 [0.745, 1.577]	0.6723		
≥5	71 (42.77)				
Tumor cellularity	0.36 (0.20)	5.280 [1.952, 14.282]	0.0010	4.287 [1.460, 12.589]	0.0081
Interaction between Lymph node metastasis and surgical margin status					0.0053
lymph node metastasis present at surgical margin status positive				0.587 [0.272, 1.266]	
lymph node metastasis present at surgical margin status negative				2.618 [1.260, 5.437]	

## Data Availability

The data that support the findings of this study are available on request from the corresponding author, (L.R.H.).

## Supplementary Materials

The following are available online at https://www.mdpi.com/2077-0383/10/3/490/s1. Figure S1. Kaplan-Meier plot of the survival probabilities of patients with high and low NFD. Figure S2. Forest plot of hazard ratios and corresponding 95% confidence limits.

## Code Availability

Source codes are available at https://github.com/janniehues/PGP-pancreas/.
