# Supplementary material for: Nerve Fibers in the Tumor Microenvironment Are Co-Localized with Lymphoid Aggregates in Pancreatic Cancer
Source: J Clin Med. 2021 Jan 30;10(3):490. doi: 10.3390/jcm10030490 (PMC7866811; doi:10.3390/jcm10030490)
Supplement: Supplementary file 1 [file jcm-10-00490-s001.pdf]

Supplementary Information

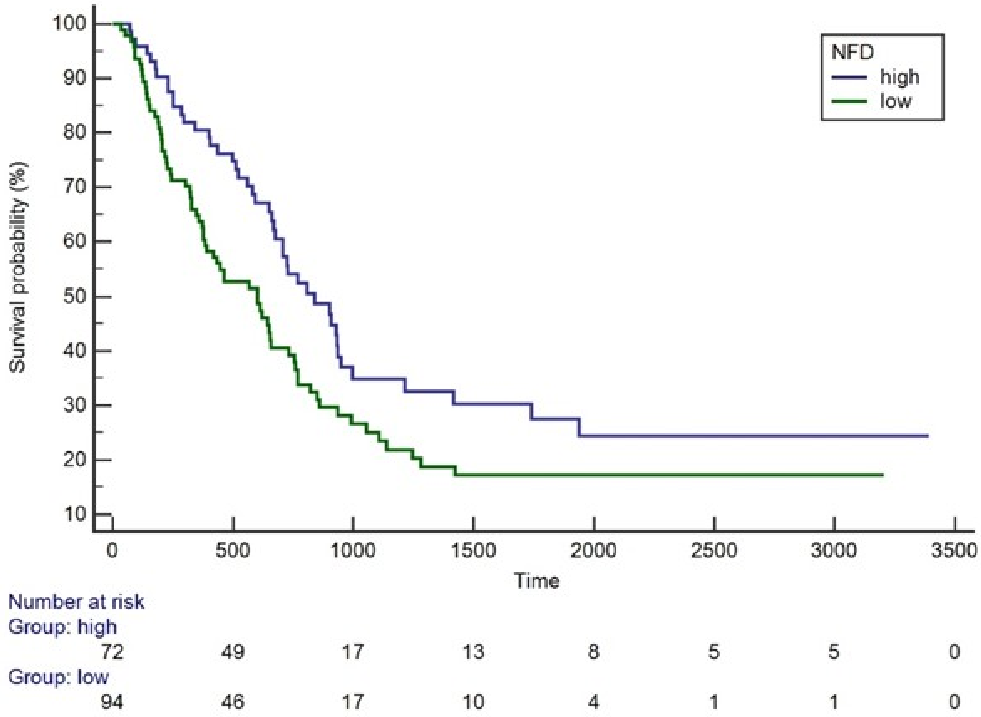

Figure S1. Kaplan-Meier plot of the survival probabilities of patients with high and low NFD.

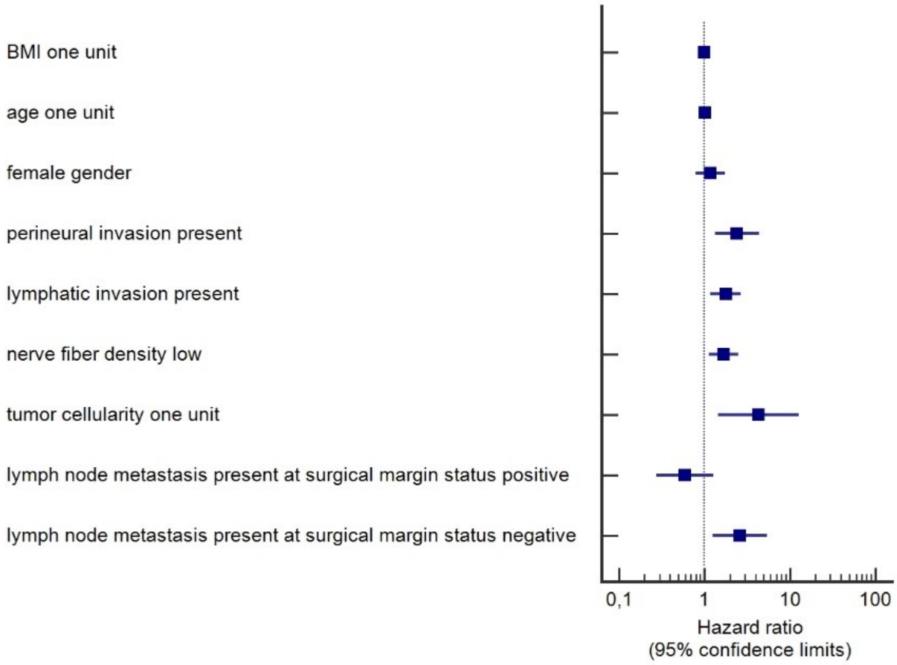

Figure S2. Forest plot of hazard ratios and corresponding 95% confidence limits.
